# Sodium glucose transporter 2 inhibitors: Will these drugs benefit non‐diabetic veterinary patients with cardiac and kidney diseases?

**DOI:** 10.1111/jvp.13472

**Published:** 2024-07-13

**Authors:** Jonathan Elliott, Mark A. Oyama

**Affiliations:** ^1^ Department of Comparative Biomedical Sciences Royal Veterinary College, University of London London UK; ^2^ Department of Clinical Sciences & Advanced Medicine University of Pennsylvania School of Veterinary Medicine Philadelphia Pennsylvania USA

**Keywords:** cardioprotection, cats, dogs, nephroprotection, tubuloglomerular feedback

## Abstract

Sodium glucose transporter type 2 (SGLT2) inhibitors have been introduced into human medicine where their beneficial effects go beyond the expected improvement in blood glucose control. These drugs appear to prevent progression of both cardiovascular and kidney diseases, not only in diabetic but also in non‐diabetic human patients. As these drugs have received conditional approval for use in diabetic cats and are being used in other veterinary species, the intriguing question as to whether they will have similar cardioprotective and nephroprotective effects in dogs and cats is being asked. The primary mechanism(s) by which SGLT2 inhibitors are cardio‐ and nephroprotective remain to be fully characterized. This paper reviews these suggested mechanisms in the context of the pathophysiology of progressive cardiovascular and kidney diseases in dogs and cats with the goal of predicting which categories of non‐diabetic veterinary patients these drugs might be of most benefit.

## 1 INTRODUCTION

Sodium glucose transporter type 2 (SGLT2) inhibitors are a new class of drug introduced in human medicine to aid control of blood glucose in diabetic patients with the first drug being approved by FDA in 2013. It soon became clear from the clinical trials that these drugs did far more than assist in blood glucose control. Type 2 diabetes (T2D) in humans is associated with a significant increased risk of cardiovascular and kidney disease resulting in progressive disease in these organ systems and premature mortality, an effect unrelated to blood glucose control. While experimental models of obesity and associated hypertension in dogs are associated with kidney pathology (Gu et al., 2004; Liu et al., 2020), based on the available evidence (mostly small retrospective studies), neither obesity nor overt diabetes mellitus in dogs and cats is associated with increased risk of incident cardiovascular or kidney disease (Greene et al., 2014; Pérez‐Sánchez et al., 2015; Tefft et al., 2014). However, this group of drugs appeared to slow progression of cardiovascular and kidney disease to defined end points in non‐diabetic as well as diabetic human patients. This raises the intriguing question as to whether these drugs might be effective in managing the different forms of cardiovascular and kidney diseases that commonly occur in cats and dogs. The aim of this review was to explore whether the mechanisms by which SGLT2 inhibitors are thought to benefit non‐diabetic human patients make them applicable as drugs to manage canine and feline cardiovascular and kidney diseases.

## 2 EVIDENCE OF NEPHROPROTECTION IN HUMAN CLINICAL TRIALS

Ma et al. (2023) undertook a systematic analysis of the four human clinical trials where non‐diabetic patients with chronic kidney disease had been recruited (DIAMOND—Cherney et al., 2020; DAPA‐CKD—Heerspink et al., 2020; et al., EMPRA—Antlanger et al., 2022; EMPA‐KIDNEY—Herrington et al., 2023). This analysis identified six groups of non‐diabetic patients from these four randomized controlled clinical trials. In total, 5416 patients were randomized to receive an SGLT2 inhibitor or placebo. These studies involved either dapagliflozin or empagliflozin and lasted between 6 weeks (cross‐over study) and 2.4 years with the majority of patients (98.6% or 5340) studied for 2 years or longer. Most patients (93.1%) were classified as non‐diabetic nephropathy patients but subgroups from one study of IgA nephropathy and focal segmental glomerulosclerosis were identified. Across these studies, compared with placebo, SGLT2 inhibitors significantly delayed the decline in eGFR (mean difference in the decline in eGFR over the study period between placebo and SGLT2 inhibitor‐treated patients was 1.35 mL/min/1.73 m^2^, 95% CI 0.84–1.80), reduced urine albumin to creatinine ratio (mean difference −24.47%; 95% CI −38.9 to −10.04) and reduced systolic arterial blood pressure (mean difference −4.13 mmHg; 95% CI −7.49 to −0.77).

Next to diabetic nephropathy, the most common predisposing conditions giving rise to CKD in human patients are hypertensive nephropathy and glomerulonephritis (Jha et al., 2013). Most patients entered into clinical trials with SGLT2 inhibitors will also have been receiving some form of renin‐angiotensin‐aldosterone blockade (RAAS blockade) suggesting any effect seen is additional to RAAS inhibition. Obesity and lifestyle factors impact on the progression of CKD whatever the underlying cause in the human population (Chesnaye et al., 2024) and so while non‐diabetic patients benefit from SGLT2 inhibitors, it is possible that the pre‐diabetic phenotype (obesity and lack of exercise; metabolic syndrome) might be part of the reason for this benefit. This may have implications for the translatability of potential mechanisms from human patients (and laboratory models) to dogs and cats, where the evidence that obesity and insulin resistance predisposes to progressive renal injury is generally lacking.

## 3 EVIDENCE OF CARDIOVASCULAR PROTECTION IN HUMAN CLINICAL TRIALS

Benefit of SGLT2 inhibitors on cardiovascular outcomes in human patients with or without concurrent T2D is firmly established. The initial finding that SGLT2 inhibitors significantly reduced morbidity and mortality in patients with T2D (Wiviott et al., 2019; Zinman et al., 2015) was followed by discovery that these benefits extended to heart failure (HF) patients without T2D. In patients with HF, SGLT2 inhibitors reduce risk of major cardiovascular (CV) morbidities, CV death and all‐cause mortality, independent of the presence of T2D. (Anker et al., 2021; McMurray et al., 2019; Packer et al., 2021; Solomon et al., 2022; Spertus et al., 2022) Meta‐analyses of the largest trials reported significantly reduced risk of cardiovascular death or hospitalization compared with placebo by 18% to 28% (Jhund et al., 2022; Vaduganathan et al., 2022), which compares favourably to other approved drugs, such as beta‐blockers, ACEi and aldosterone receptor antagonists. The success of these and other studies form the basis for approval of SGLT2 inhibitors for treatment of HF in the United States and other countries. At the time of writing, SGLT2 inhibitors are a Class 1 (i.e., strong) recommendation for human patients with reduced ejection fraction and Class 2a (i.e., moderately strong) recommendation for HF patients with mildly reduced or preserved ejection fraction. (Heidenreich et al., 2022) Two properties of SGLT2 inhibitors in clinical trial patients bear mention. First, a significant reduction in risk is noted as soon as 28 days after initiation of SGLT2 inhibitor treatment, which is a relatively rapid effect compared to other chronic medications (Butler et al., 2020). Second, SGLT2 inhibitors have a remarkably high safety and tolerability margin. Severe adverse effects such as hypotension, acute kidney injury and electrolyte imbalances are rare (McMurray et al., 2019; Packer et al., 2021). The success of SGLT2 inhibitors in human patients with HF fuels interest in veterinary medicine.

### 3.1 Pharmacological effects of SGLT2 inhibitors and their impact on cardiovascular and kidney physiology

#### 3.1.1 Diuretic effects of SGLT2 inhibitors

The diuretic mechanism of SGLT2 inhibition is distinct from loop diuretics in their site of action within the nephron, target solute carrier and pleotropic effects. The SGLT2 carrier is responsible for transporting 90% of the filtered glucose in the proximal convoluted tubule (PCT). Treatment with SGLT2 inhibitors presents more sodium and glucose with its attendant water to the S3 segment of the PCT where SGLT1 transporters are expressed. Most SGLT2 inhibitors are selective for SGLT2 and have much lower affinity for SGLT1 transporters (Hummel et al., 2012; Lu et al., 2014) which are also upregulated and reclaim some of the sodium and glucose escaping reabsorption in the first two PCT segments. Nevertheless, a good proportion overflows into the later parts of the nephron. Non‐selective SGLT inhibitors which block both SGLT1 and SGLT2 have been developed and inhibit glucose uptake in the kidney and intestine (e.g., Sotagliflozin; Iyer et al., 2023). Most published data in human medicine involves drugs which selectively inhibit SGLT2 and data from head‐to‐head comparisons between these drugs and sotagliflozin are not available. Sotagliflozin shows similar cardioprotective effects of in human clinical trials (Bhatt et al., 2021).

By inhibiting sodium reabsorption in the PCT, activation of the tubuloglomerular feedback (TGF) mechanism (see below) will occur leading to local generation of adenosine which constricts the afferent arteriole. Adenosine is generated locally within the juxtaglomerular apparatus in response to the high NaCl detected by the macula densa and constricts the afferent arteriole by stimulating A1 adenosine receptors (Schnermann & Briggs, 2008). SGLT2 is co‐expressed with the sodium hydrogen exchange (NHE3) in the PCT. SGLT2 inhibitors also inhibit the activity of this transporter, reducing a second pathway for sodium reabsorption (Onishi et al., 2020). This mechanism explains the well‐recognized decrease in eGFR seen in human patients when starting SGLT2 inhibitors.

The diuretic effect of SGLT2 inhibitors is associated with less activation of the RAAS than with other diuretic drugs. In part, this is because the TGF mechanism inhibits renin release whereas loop diuretics, such as furosemide, inhibit chloride sensing in the macula densa and activate renin release (Beierwaltes, 1995). In addition, inhibition of glucose reabsorption means that water is held in the tubule whilst the distal nephron segments reabsorb sodium to compensate for the deficient PCT reabsorption of sodium. However, ultimately free water clearance is reduced because of vasopressin secretion driven by an increase in plasma sodium concentration (Masuda et al., 2020). This maintains circulating fluid volume and contrasts with the action of furosemide, which decreases circulating fluid volume and activates RAAS (Masuda et al., 2022).

#### 3.1.2 Effects of SGLT2 inhibitors on sympathetic nerve activity

Reciprocal cross‐talk between SGLT2 and sympathetic nerve activity occurs such that stimulation of the renal sympathetic nerves leads to upregulation of PCT SGLT2 (Matthews et al., 2017) and SGLT2 inhibitor treatment reduces sympathetic nerve activity (Herat et al., 2020). The precise physiological mechanism(s) by which this cross‐talk occurs are unknown but the importance of sympathetic nerve activity in cardiovascular and kidney diseases and the associated hypertension is well recognized in human medicine and under‐investigated in veterinary medicine.

#### 3.1.3 Effects of SGLT2 inhibitors on other membrane ion transporters

In addition to their inhibitory effects on SGLT2 transporters, which appear to be only expressed in the PCT of the kidney, SGLT2 inhibitors also inhibit sodium hydrogen ion exchangers (NHEs) which transport sodium ions into the cell and hydrogen ions out (Baartscheer et al., 2017; Uthman et al., 2018). NHE‐1 proteins are expressed in monocytes, endothelial cells and cardiomyocytes and have a complex role in regulating cell function through modulation of intracellular pH and calcium (see Boedtkjer & Aalkjaer, 2012). Upregulation and over‐activity of NHE1 in vascular cells and cardiac myocytes and the potential benefits of SGLT2 inhibitors on cardiovascular diseases are discussed below.

#### 3.1.4 Effects of SGLT2 inhibitors on metabolism at the whole organism level

Nutrient excess programmes cells for growth by activating the Akt (protein kinase B)/mammalian target of rapamycin (mTOR) pathway which regulates hundreds of genes promoting anabolism, inhibiting autophagy and upregulating mitochondrial production of ROS. Autophagy is important for intracellular homeostasis, leading to controlled degradation of proteins and organelles (within autophagosomes) making cells resilient to stressors like hypoxia and starvation.

The counter‐balancing pathways to Akt/mTOR, which prepare cells for stress of nutrient and oxygen deprivation, are the sirtuins and adenosine monophosphate‐activated protein kinases (AMP‐kinase) pathways. Sirtuins are redox‐sensitive NAD‐dependent histone deacetylases, activation of which alleviates oxidative and reticulo‐endoplasmic stress, facilitates disposal of dysfunctional mitochondria and promotes mitochondrial biogenesis (Sun et al., 2023; Zhang et al., 2024). PGC‐1α (peroxisome proliferator‐activated receptor γ coactivator 1‐α) is a downstream effector in the sirtuin‐induced mitochondrial biogenesis pathway. AMP‐kinase partners with the sirtuins in counterbalancing the Akt/mToR pathway. AMP‐kinase is activated when the cellular AMP:ATP ratio is high, thus sensing energy deprivation.

Packer (2022) proposed that SGLT2 functions as an energy sensor, identifying an excess nutrient state. Thus, SGLT2 inhibitors mimic nutrient deprivation at the whole organism level. In the human clinical trials, treatment with SGLT2 inhibitors stimulates ketogenesis (Ekanayake & Mudaliar, 2022) and erythrocytosis (Gangat et al., 2021; Mazer et al., 2020; Zinman et al., 2015), which mimic responses to nutrient deprivation and hypoxia. Packer (2020) argues that increasing production of ketone bodies and red cells indicates that SGLT2 inhibitors have activated the body's response mechanism to nutrient deprivation and hypoxia in general, including in cells that do not express SGLT2. Osataphan et al. (2019) demonstrated that SGLT2 inhibitor administration to mice caused a metabolic switch away from glucose utilization towards fatty acid oxidation mimicking a fasting state, an effect that was associated with downregulation of mTOR1 and upregulation of AMP‐kinase in liver tissue. Such a systemic nutrient deprivation effect of SGLT2 inhibitors could occur because of a direct interaction of the drugs with sirtuin‐1 (encoded by the SIRT‐1 gene) or one of its downstream effectors (AMP‐kinase or PGC1α; see Packer, 2020, 2022). Canagliflozin increased AMP‐kinase activity via an inhibitory effect on respiratory complex I (Hawley et al., 2016), a finding which supports this suggestion.

**FIGURE 1:**
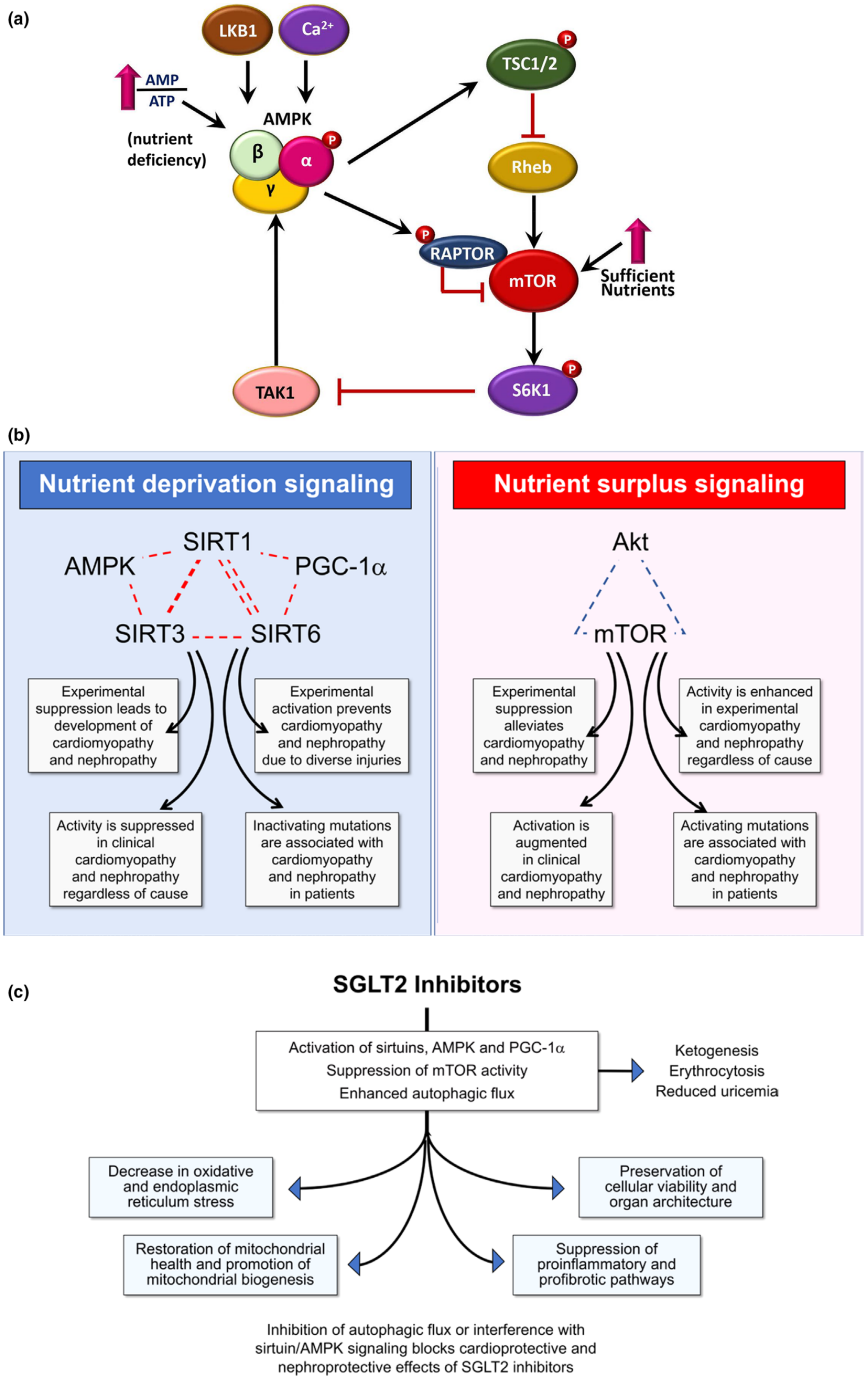
(a) Brief summary of reciprocal regulation between AMPK and mTOR pathways. AMPK is activated by upstream signalling pathways, including LKB1, CaMKKII and TAK1. AMPK pathway inhibits mTOR signalling, through at least two mechanisms, that is, phosphorylation of TSC2 via Rheb, and direct phosphorylation of RAPTOR. In addition, mTOR‐S6K1 signalling pathway inhibits AMPK activation through inactivation of TAK1. Adapted from Jo et al. (2019). (b) Effect of nutrient deprivation and nutrient surplus signalling on the evolution and progression of cardiomyopathy and nephropathy in experimental and clinical settings. Reproduced from Packer (2022). (c) Proposed framework by which SGLT2 (sodium‐glucose co‐transporter 2) inhibitors can modulate nutrient deprivation signalling and thereby enhance autophagic flux and reduce cellular stress. (Reproduced from Packer, 2022). Akt, protein kinase B; AMPK, adenosine monophosphate‐activated protein kinase; LKB1, liver kinase B1 (also known as STK11); mTOR, mammalian target of rapamycin complex; PGC‐1α, peroxisome proliferator‐activated receptor γ coactivator 1‐α; RAPTOR, regulatory associated protein of mTOR; Rheb, Ras (GTPase) homologue enriched in brain; S6K1, ribosomal protein S6 kinase B1; SIRT1, sirtuin 1; SIRT3, sirtuin 3; SIRT6, sirtuin 6; TAK1, TGF‐β‐activated kinase 1; TSC1/2, disease genes for tuberous sclerosis complex 1/2.

These three major regulators of cell physiology, namely Akt/mTOR and sirtuins/PGC1α with AMP‐Kinase are highly interconnected molecularly (see Packer, 2022; see Figure 1) regulating each other. Upregulation of Akt/mTOR will lead to inhibition of sirtuins/PGC1α and/or AMP‐kinase and vice versa. Equally, disease can disturb the balance maintaining homeostasis of cellular bioenergetics and leave the cells susceptible to stress. Tipping the balance in favour of the Akt/mTOR pathway inhibits autophagy, a process which allows degradation of dysfunctional organelles, such as mitochondria and peroxisomes, and large protein complexes. Autophagy generates energy for the cell, prevents excess ROS production, inhibits apoptosis (Inoki et al., 2021) and markedly reduces proinflammatory and profibrotic processes (Levine et al., 2011).

The fact that SGLT2 inhibitors promote erythrocytosis (Mazer et al., 2020) suggests that they exert effects via hypoxia‐inducible factors (HIFs) (see Packer, 2021). Two isoforms of HIF, HIF1α and HIF2α are upregulated by hypoxia. In the presence of adequate oxygen tension, they are enzymatically hydroxylated (pyrolyl hydroxylases), ear‐marking them for degradation, so preventing them from combining with HIF1β and influencing the transcription of many genes, including *EPO*. HIF2α is the isoform produced by renal pericytes that influences *EPO* production. HIF1α's action is primarily to upregulate genes encoding enzymes and transporters that reduce oxygen use and promotes mitophagy (removal of dysfunctional mitochondria; a source of ROS), which are beneficial in hypoxic stress. However, with chronic activation in kidney disease, HIF1α is proinflammatory and profibrotic (see below; Kong et al., 2017). HIF2α promotes the disposal of dysfunctional peroxisomes (another source of ROS) and upregulates erythropoietin. Experimental evidence suggests that SGLT2 inhibitors lead to upregulation of HIF2α and downregulation of HIF1α (see below).

#### 3.1.5 Metabolic switch driven by response to SGLT2‐mediated osmotic diuresis

Loss of glucose in urine following SGLT2 inhibition leads to mobilization of glucose stores and the synthesis of glucose from proteins and glycerol (gluconeogenesis). This metabolic switch generates ketone bodies, such as β‐hydroxybutyrate and acetoacetate, fuels which are utilized by the kidney and cardiac tissue in preference to glucose.

An adaptive response to the osmotic diuresis resulting from SGLT2 inhibition adds to the generation of ketone bodies. This compensatory response has been compared with the water‐conserving mechanism activated in aestivating lung or cartilaginous fish in high‐salinity marine environments (Marton et al., 2021). Studies in human patients and rats demonstrate that adaptive responses reduce free water clearance during treatment, an effect mediated by increased vasopressin secretion as discussed above (Eickhoff et al., 2019; Masuda et al., 2020). Vasopressin increases urea transporter expression in the nephron (Yang & Bankir, 2005). To mount this response requires the liver to make more urea, an energy‐intensive process requiring either an increase in energy intake and/or exploitation of protein reserves from skeletal muscle. Catabolism of amino acids from skeletal muscle generates ammonia which, by the process of transamination yields alanine from pyruvate. At the liver, this cycle is reversed and alanine used to generate ammonia for urea production and pyruvate for gluconeogenesis (see Marton et al., 2021). If a state of negative energy balance results, the liver will produce more ketones from fatty acid metabolism. In addition, branched chain amino acid catabolism occurs within the kidney and heart with transaminases producing alanine (from pyruvate) for the liver to use to make urea. The resulting branched chain α‐keto‐acids become available energy sources for these organs (Kappel et al., 2017). Thus, the loss of glucose (energy) and loss of water (osmotic diuresis) stimulated by SGLT2 inhibitors leads to a physiological adaptation that combines generation of alternative energy sources (glucose from gluconeogenesis and ketones) and limits loss of water (Marton et al., 2021).

## 4 CONCLUSIONS TO THE PHARMACOLOGICAL EFFECTS OF SGLT2 INHIBITORS

**FIGURE 2:**
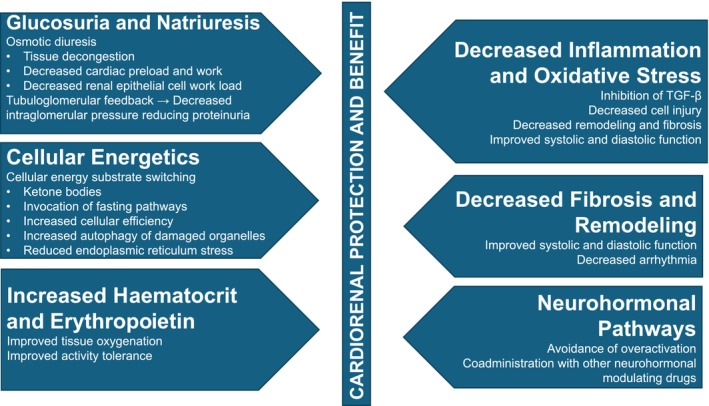
Summary of the major potential cardiorenal protective mechanisms related to actions of SGLT2 inhibitors.

From the above discussion of the many pharmacological effects of SGLT2 inhibitors, the mechanisms by which SGLT2 inhibitors exert their effects are numerous and related to both glucose reduction or independent of glucose (see Figure 2). To what extent each benefits patients with HF and/or kidney disease is incompletely understood. Many of the proposed mechanisms might apply to dogs and cats with naturally occurring heart failure and kidney disease, therefore studies in veterinary species are of interest. The next part of this review will present various proposed mechanisms of SGLT2 inhibitors in the setting of heart and kidney diseases, first considering proposed mechanism by which these drugs benefit human patients and then discussing whether these benefits could be extrapolated to veterinary patients.

## 5 PROPOSED SGLT2 INHIBITOR MECHANISMS IN HEART FAILURE AND CARDIOVASCULAR DISEASE

Despite substantial evidence of clinical benefit, the mechanisms associated with SGLT2 inhibition in HF are incompletely understood and likely multifactorial. Proposed benefits include reduction in blood volume, protection against myocyte remodelling, oxidative stress and inflammation, neurohormonal alterations, shifts in energy substrate utilization and changes in the cardiorenal axis. (Sullivan et al., 2022; Verma et al., 2024; Zhang et al., 2023) Many of the beneficial mechanisms relevant to HF are related to the glucose‐lowering effect while others are indirectly or only tangentially related. For instance, SGLT2 inhibitors can affect the myocyte‐specific Na‐H exchanger (NHE1), which might help explain its many and varied effects (Verma et al., 2024). The pleotropic effects of SGLT2 inhibition not only offer a chance to better understand the effect of this class of drug on HF, but also the pathophysiology of HF itself, which can lead to further advances.

### 5.1 Reduction in intravascular and extravascular fluid

Glucosuria increases urine volume through osmotic diuresis and helps reduce signs of congestion and myocardial workload. The fluid loss induced by SGLT2 inhibition might be particularly effective at reducing extravascular fluid in the lungs and other organs through its effect on Na‐H balance in the PCT and an increase in electrolyte‐free water clearance (EFWC) (de la Espriella et al., 2021; Hallow, Greasley, et al., 2018; Hallow, Helmlinger, et al., 2018) This mode of action coupled with proposed sequestering of osmotically inactive Na that is left behind in peripheral tissues (possibly bound to glycosaminoglycans in skin, muscle and bone) is particularly effective at reducing the amount of interstitial water necessary to equilibrate with interstitial Na. Modelling of the Na and water content of the intravascular, extravascular and peripheral spaces suggests that SGLT2 inhibition removes three times as much water from the interstitial space as compared to the intravascular space while loop diuretics removed only 2/3 the amount of interstitial fluid as compared to blood volume (Hallow, Helmlinger, et al., 2018). Thus, SGLT2 inhibitors appear particularly adept at shifting fluid of out the interstitial space while preserving arterial filling and blood pressure, which avoids excessive neurohormonal activation as compared to traditional loop diuretics (Shiina et al., 2023). The preservation of intravascular volume might explain the high safety and tolerability profile of SGLT2 inhibitors with respect to blood pressure and kidney perfusion. This might also explain the absence of decreases in natriuretic peptide concentrations, which are a marker of intracardiac and intravascular volume, whereas proposed markers of interstitial fluid, such as carbohydrate antigen 125, are decreased in human patients (de la Espriella et al., 2021). In patients receiving SGLT2 inhibitors, prevention of further increases in natriuretic peptide concentration over time might be more likely than a marked reduction in values (Ibrahim & Januzzi, 2021; Lawler et al., 2021; Packer et al., 2021; Tanaka & Node, 2020). Based on our current understanding of the pathophysiology of congestive HF, it seems likely that these differential effects of SGLT2 inhibitors on interstitial versus intravascular fluid volumes will translate to dogs and cats with HF.

### 5.2 Increased haematocrit and erythropoietin (EPO) production

Anaemia is a common comorbidity in human patients with HF and a strong predictor of morbidity, such as poor activity tolerance, and mortality. Renoprotection from SGLT2 inhibition is postulated as protecting the kidney against hypoxia and oxidative stress, which disrupts EPO production (see below). SGLT2 inhibitor treatment is associated with increased haematocrit, red blood cell count and improved tissue oxygen delivery, independent of haemoconcentration secondary to diuresis (Ekanayake & Mudaliar, 2023). In addition to stimulating EPO production, SGLT2 inhibition is associated with suppression of iron‐inhibiting inflammatory and anaemia‐related proteins, such as hepcidin. These actions of SGLT2 inhibition increase reticulocyte count, mobilize iron stores and increase ferritin, leading to a sustained increase in haematocrit even after plasma volume re‐equilibrates in the weeks and months after initiation of treatment (Ekanayake & Mudaliar, 2023; Ghanim et al., 2020). The effects of SGLT2 inhibition on endogenous EPO and haematocrit might confer benefit as opposed to exogenous EPO, which can cause thrombotic events and is not recommended. Haematocrit can be viewed alongside clinicopathologic measures of hydration and fluid status to help differentiate increased red blood cell production from haemoconcentration in the clinical setting. Haematocrit is easily monitored and, in at least one human patient study, increased haematocrit was the strongest predictor of risk reduction following SGLT2 inhibitor administration (Inzucchi et al., 2018).

### 5.3 Cardiac antifibrotic and anti‐remodelling effects

SGLT2 inhibitors exhibit antifibrotic properties in the cardiac interstitium and vasculature (Bonanni et al., 2023). Fibrosis is characterized by excessive extracellular matrix deposition and is a hallmark of adverse cardiac remodelling that occurs in response to a host of different primary cardiac diseases. In human HF patients, cardiac magnetic resonance imaging demonstrated significantly decreased extracellular matrix volume in patients randomized to empagliflozin versus placebo (Requena‐Ibanez et al., 2021). These changes were accompanied by suppression of inflammatory markers, such as tumour necrosis factor. Another key inducer of fibrosis in cardiac tissues is transforming growth factor β (TGF‐β) and inhibition of TGF‐β‐mediated hypertrophic signalling pathways is viewed as a key mechanism by which SGLT2 inhibitors confer protection against cardiac structural and functional changes (Bonanni et al., 2023; Chen et al., 2022). SGLT2 inhibitors further influence the cardiac ECM by modulating its synthesis and degradation. Adjustment of the balance of matrix metalloproteinases (MMPs) and tissue inhibitors of metalloproteinases (TIMPs) prevents excessive matrix turnover (Kang et al., 2020; Ortega‐Paz et al., 2023). Preservation of cardiac tissue architecture and reduction in fibrosis contribute to improved myocardial contractility and relaxation.

The antifibrotic effect of SGLT2 inhibitors might be particularly relevant to cardiac diseases in both humans and veterinary species characterized by substantial fibrosis, including many of the arrhythmogenic cardiomyopathies (Yang et al., 2022). SGLT2 inhibitors might further directly affect myocyte function by blockade of sarcolemmal NHE1, which protects myocytes from Na and Ca overload, which can trigger arrhythmias. Activation of pro‐hypertrophy and pro‐fibrosis pathways accompany the overloaded HF phenotype in experimental models (Baartscheer et al., 2017) but the uniformity of these effects and applicability to humans and veterinary species require further study (Baek et al., 2013; Chung et al., 2021).

### 5.4 Anti‐inflammatory effects and reduction of oxidative stress

The role of the inflammatory milieu or inflammasome in HF has been a subject of growing interest (Murphy et al., 2020). Inflammation, whether arising from comorbidities or as direct release of proinflammatory cytokines by injured vascular and myocardial cells, can be wide ranging, and injurious to other organ systems including the kidneys and GI tract. SGLT2 inhibitors reduce epicardial and myocardial adipose tissue, a potential source of inflammatory cytokines during heart disease (Requena‐Ibanez et al., 2021). Oxidative stress, which is closely related to inflammation, is also is a key driver of cardiovascular remodelling (Wang & Kang, 2020). Oxidative stress negatively affects cardiomyocyte mitochondrial function, vascular smooth muscle and ventricular function (Munzel et al., 2017). SGLT2 inhibitors have anti‐inflammatory and antioxidative properties that attenuate inflammatory signalling cascades, such as TGF‐β, C‐reactive protein, interleukin‐6 and NHE1 pathways in diseased myocytes, implicated in adverse cardiac remodelling and fibrosis (Bendotti et al., 2023; Lee et al., 2022; Zhang et al., 2023). Mitigation of inflammation and oxidative stress contributes to the preservation of vascular and cardiac structure. Vascular endothelial function can be specifically improved by SGLT2 inhibition through promotion of nitric oxide (NO) mediated bioavailability and vasodilation (see Li et al., 2022). Enhanced NO production may counteract vascular remodelling, endothelial dysfunction, inflammation and oxidative stress. The benefits of SGLT2 inhibition might further extend to the gut microbiome and suppression of bacterial species that produce cardiotoxic or inflammatory metabolites (Billing et al., 2024).

In veterinary medicine, assays exist for many different inflammatory markers and signals, and studies in dogs with HF point to the presence of a proinflammatory state (Di Loria et al., 2024; Domanjko Petrič et al., 2018; Nemec Svete et al., 2021). In both humans and veterinary species, the complexity and interrelatedness of the different inflammatory and oxidation pathways presents a challenge, but further studies would help better define and specify the effects of SGLT2 on the most important aspects.

### 5.5 Modulation of neurohormonal pathways

SGLT2 inhibitors impact various neurohormonal systems, including the RAAS and sympathetic nervous system. Both are activated in HF and contribute to the pathology and progression of disease. RAAS inhibition leads to vasodilation, reduced sodium retention and decreased cardiac workload. Attenuation of sympathetic tone lowers heart rate and improves cardiac efficiency. The effects of SGLT2 inhibitors on RAAS and autonomic tone likely are mediated indirectly, through change in blood volume, kidney perfusion and vasomotor tone, as well as directly by interfering with intrarenal neurohormonal systems (Puglisi et al., 2021). An important distinction between SGLT2 inhibitors and traditional diuretics, such as furosemide, is the reduced tendency for SGLT2 inhibitors to trigger RAAS activity through intravascular volume depletion and tubuloglomerular feedback (see above). It is difficult to ascertain a consistent effect of SGLT2 inhibitors on neurohormonal pathways in clinical patients. In practice, SGLT2 inhibitors are frequently co‐administered with RAAS or sympathetic nervous system inhibiting drugs, such as ACE inhibitors, mineralocorticoid antagonists and β‐blockers, which likely provides comprehensive neurohormonal control (Heidenreich et al., 2022).

### 5.6 Alteration of myocardial energetics and metabolism

The heart is a metabolically demanding organ and processes a variety of substrates to meet its energy demands. It is metabolically flexible in that it can preferentially utilize glucose, fatty acids, ketones or amino acids depending on the circumstances (Actis Dato et al., 2024). In human patients with HF, glycolysis becomes the primary energy source but is associated with increased oxidative stress (Chen, Zou, et al., 2023). The urinary glucose excretion associated with SGLT2 inhibition results in lowered plasma glucose levels and improved insulin sensitivity. Enhanced glucose uptake by peripheral tissues, including cardiac muscle cells, contributes to more efficient energy utilization as does mobilization of epicardial and visceral fat stores, and increased glucagon levels (Saucedo‐Orozco et al., 2022). SGLT2 inhibitors have been postulated to induce a shift in energy metabolism towards utilization of fatty acids and ketone bodies, such as β‐hydroxybutyrate (Chase et al., 2023). This phenomenon, known as ketosis, provides an alternative cardiac energy substrate. The myocardium might benefit from increased reliance on ketone bodies, which are more oxygen‐efficient than fatty acids, leading to improved myocardial energetics (Pherwani et al., 2024). The reduction in available glucose might also induce a protective fasting state that increases autophagic housekeeping of damaged or unnecessary organelles and proteins (see above), which increases cellular efficiency and reduces oxidative stress (Packer, 2023). This thrifty substrate or ‘super fuel’ hypothesis, while still controversial (Osto et al., 2023; Packer, 2022, 2023; Voorrips et al., 2023), might trigger protective fasting signalling pathways that maintain mitochondrial health and function, increase autophagic degradation of unwanted organelles and suppress oxidative stress and inflammation (Ferrannini et al., 2016; Packer, 2020). Metabolic changes occur relatively rapidly in response to SGLT2 inhibition and might explain the observation of early benefit before substantial cardiac remodelling has time to occur in human patients (Cowie & Fisher, 2020). In dogs with heart disease, mitochondrial fatty acid oxidation, glycolysis and ketone body utilization are disrupted (Freeman et al., 2023; Li et al., 2021) opening the possibility of benefit from SGLT2 inhibitors. Assessment of myocardial metabolism is challenging. The presence of altered energetics using a range of diagnostic methods is inconsistent and controversial (Ferrannini et al., 2016; Hundertmark et al., 2023; Inzucchi et al., 2018; Santos‐Gallego et al., 2023), which might hinder targeted and specific investigation in veterinary studies.

## 6 SGLT2 INHIBITORS IN HEART FAILURE WITH PRESERVED EJECTION FRACTION

In human patients, heart failure with preserved ejection fraction (HFpEF) is responsible for approximately half of all HF cases (Faluk et al., 2024). The term HFpEF is used to describe HF in the presence of normal ejection fraction (i.e., systolic function). While the comparison is not perfect, cats with hypertrophic or restrictive cardiomyopathy display clinicopathological features, such as dyspnoea, poor diastolic function and myocardial fibrosis, similar to human patients with HFpEF. Treatment for HFpEF is poorly understood as compared to treatment of HF due reduced ejection fraction and poor contractility. An intriguing aspect of SGLT2 inhibition is the potentially beneficial effects in HFpEF, and by extension, interest in veterinary species with related conditions. Many of the previously described mechanisms related to SGLT2 inhibition in HF, including reducing fibrosis, improving myocardial energetics, and cardiorenal axis protection are believed to apply to HFpEF (Faluk et al., 2024; Ostrominski & Vaduganathan, 2024). The hallmark of HFpEF is increased myocardial stiffness, which might be alleviated by SGLT2 inhibitors through anti‐inflammatory and antioxidative mechanisms that increase vasodilatory and anti‐remodelling substances. For example, NO can restore phosphorylation of sarcomeric proteins such as titin and enhance relaxation (Pandey et al., 2023). In human patients with HFpEF, the combined outcome of trials conducted to date revealed reduction in CV hospitalizations and death and improvement in clinical signs (Anker et al., 2021; Nassif et al., 2021; Solomon et al., 2022). Guidelines in human medicine contain a moderately strong recommendation for SGLT2 inhibitor use in HFpEF while evidence to support this continues to accumulate.

## 7 POTENTIAL RELEVANCE OF SGLT2 inhibitors IN VETERINARY SPECIES WITH HF

The natural history of HF in human patients and veterinary species have many clinicopathologic similarities, foremost of which are the mechanisms and clinical signs of congestion, cardiac remodelling and efficacy of drugs such as diuretics, ACEI and aldosterone antagonists. While the aetiology of HF with respect to coronary artery disease substantially differs in humans vs. veterinary species, many myocardial diseases, such as idiopathic dilated, hypertrophic and restrictive cardiomyopathy are common across species. The HF phenotype, involving neurohormomal activation, myocardial energy substrate usage, proinflammatory and pro‐fibrosis signalling, and cardiovascular remodelling, likely is a final common pathway, regardless of upstream aetiology. Drugs targeting these pathways should address many different HF aetiologies. Studies of SGLT2 inhibition in mice, rats and pigs with wide variety of experimental injury, demonstrate improved myocardial function, decreased filling pressure, reduced fibrosis and decreased proinflammatory and remodelling signalling molecules, suggesting that SGLT2 inhibitors might translate well to naturally occurring disease in companion animals (Panico et al., 2023). The need for new treatments in veterinary species with heart disease is clear insofar as survival times of dogs and cats with HF treated with standard therapy are relatively short. Aspects of veterinary HF that might particularly benefit from SGLT2i include the diastolic heart diseases, such as restrictive and hypertrophic cardiomyopathy, for which effective treatments are lacking, diuretic and natriuretic resistance in animals receiving traditional loop diuretics and addressing often overlooked inflammatory and oxidative stress pathways that likely contribute to disease progression.

## 8 PROPOSED MECHANISMS OF NEPHROPROTECTION BY SGLT2 INHIBITORS

Many of the actions of SGLT2 inhibitors discussed above have been proposed as protecting the kidney in both the diabetic and non‐diabetic patient. Some (protecting against oxidative stress, reducing inflammation and fibrosis) are common to the cardioprotective actions mentioned above.

### 8.1 Renal haemodynamics and tubuloglomerular feedback

In 1981, Brenner and colleagues published paper on the adaptation of remaining functioning nephrons to nephron loss (Hostetter et al., 1981). This seminal paper provided an explanation of intrinsic progression of kidney diseases through maladaptive hyperfiltration of remaining functioning nephrons leading to proteinuria and continued intrinsic nephron damage. Coined ‘the intact nephron hypothesis’, it is relevant to one proposed mechanism of action of SGLT2 inhibitors.

**FIGURE 3:**
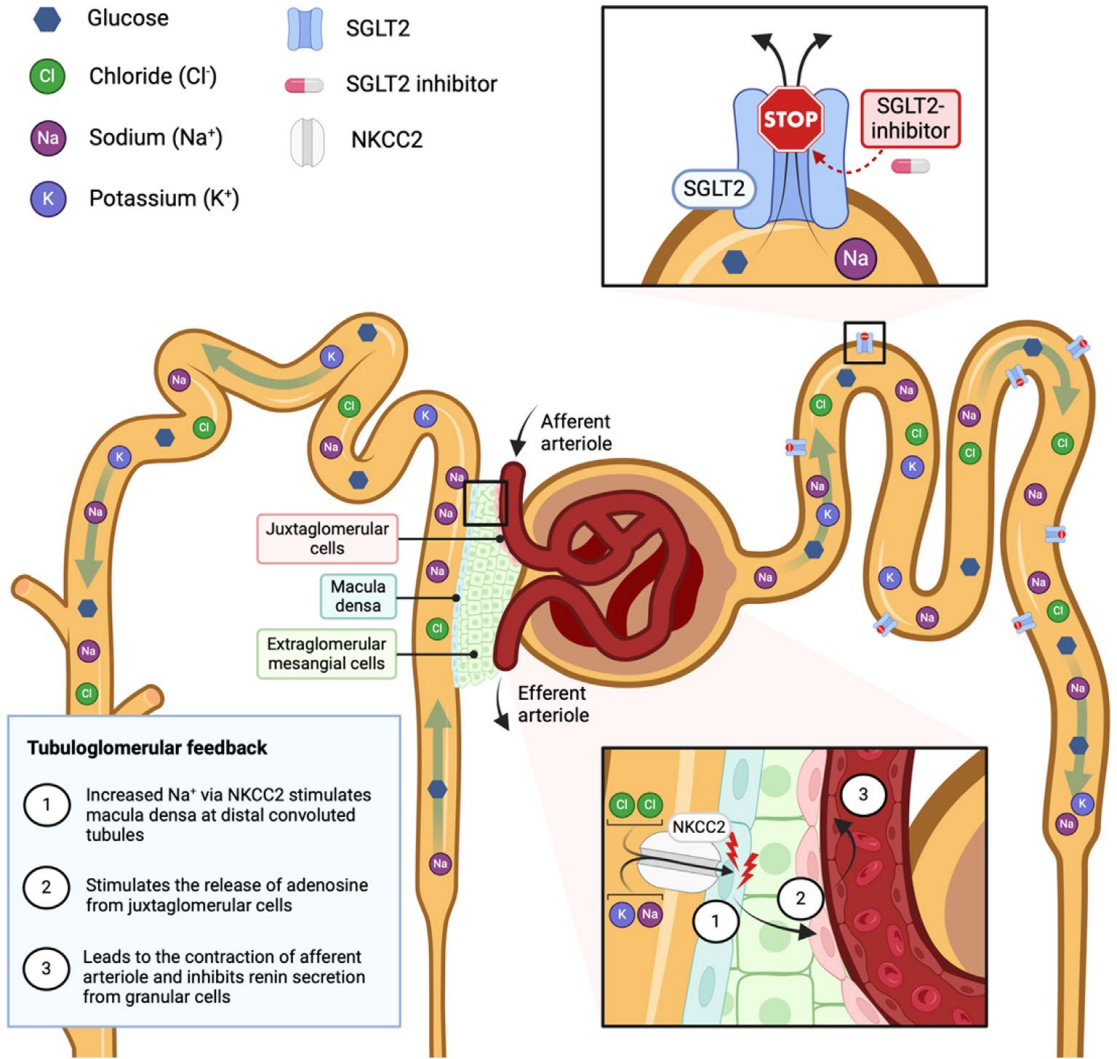
Schematic representation of tubuloglomerular feedback resulting from the inhibition of sodium and glucose reabsorption in the proximal convoluted tubule. Created using Biorender® by Pak Kan Tang.

Inhibition of sodium reabsorption in the PCT presents more sodium to the distal nephron. Sodium and chloride are sensed by the Na‐K‐Cl co‐transporter in the first part of the distal tubule (the macula densa) which signals to the afferent arteriole leading to its constriction, thus lowering glomerular filtration pressure and reducing GFR. This tubuloglomerular feedback (TGF) ensures the tubule is presented with a stable amount of filtrate. When filtrate flow is too fast, the macula densa generates adenosine (see above) which constricts the afferent arteriole, lowering downstream filtration pressure. When fluid flow is too low, macula densa cells switch off adenosine release and signal to the afferent arteriole to secrete renin, leading to afferent arteriolar dilation (reduced adenosine) and efferent arteriolar constriction mediated by angiotensin II (Kishore et al., 2018; see Figure 3).

Hyperfiltration‐associated proteinuria, secondary to glomerular capillary hypertension, accompanies many different forms of kidney disease where TGF no longer overcomes the afferent arteriolar dilation and RAAS activation that accompanies nephron damage (see Brown, 2013). RAAS inhibition lowers glomerular capillary pressure and proteinuria in diabetic and non‐diabetic forms of human CKD, reducing incident CKD in patients at risk and slowing progression in those with established CKD (see Siamopoulos & Kalaitzidis, 2008). However, the robustness of these clinical trials has been called into question recently (Garcia Sanchez et al., 2022).

Inhibition of sodium reabsorption in the first part of the PCT by SGLT2 inhibitors, activates the TGF mechanism leading to adenosine‐mediated afferent arteriolar constriction and inhibition of renin release. SGLT2 inhibitors also inhibit the sodium hydrogen exchange (NHE3) co‐expressed with SGLT2, further reducing PCT sodium reabsorption (Onishi et al., 2020). This mechanism explains the well‐recognized decrease in eGFR seen in human patients when SGLT2 inhibitors are started. Subsequently, the rate of change in eGFR is slowed by SGLT2 inhibitor treatment.

Chen, Delić, et al. (2023) demonstrated that this mechanism operated in the rat nephrectomy model. Following 5/6 nephrectomy to induce CKD, the urine adenosine to creatinine ratio was substantially reduced. Empagliflozin led to a dose‐dependent decrease in urine albumin to creatinine ratio and increased urinary adenosine excretion; the latter effect was negatively correlated with remnant kidney fibrosis (*r* = −.45; *p* = .004) and positively correlated with GFR (*r* = .36; *p* = .023) over the 95‐day study. Not all non‐diabetic models of CKD demonstrate a beneficial effect of SGLT2 inhibitors (Hojná et al., 2022). Indeed, other studies using the rat 5/6 nephrectomy model have not seen a benefit (Rajasekeran et al., 2018; Zhang et al., 2016). Differences between studies might include the strain of rat (Wistar rats being more sensitive than Sprague Dawley), dietary sodium (lower sodium intake favouring SGLT2 inhibitor beneficial effects) and whether the rats are fed ad lib or undergo a daily period of fasting (fasting assisting SGLT2 inhibitor action).

Both dog and cat kidneys respond to renal mass reduction by hyperfiltration leading to increased urinary protein loss (Brown et al., 1990; Brown & Brown, 1995). Treatment of the dog remnant kidney model with enalapril lowered glomerular capillary pressure, proteinuria and reduced remnant kidney pathology (Brown et al., 2003). Treatment of the cat model with benazepril also lowered glomerular capillary pressure and arterial blood pressure without affecting GFR but had no impact on remnant kidney pathology after 6 months (Brown et al., 2001). No studies have examined the effect of SGLT2 inhibitors in dog or cat remnant kidney models, however, the administration of dapagliflozin (1.25 mg/kg daily for 5 days) to healthy cats led to glucosuria and an unexpected increase in GFR (Gal et al., 2020). The authors of this paper postulated that cats treated with dapagliflozin compensated by upregulating sodium and chloride reabsorption in the later parts of the tubule, and so the TGF mechanism did not operate to lower GFR. Such a compensatory mechanism seems unlikely to be possible in cats with CKD although this needs to be determined by direct study in the future.

Naturally occurring canine and feline CKD epidemiology is poorly characterized. Dogs are more likely than cats to have protein‐losing diseases. Glomerular disease is the predominant form of canine CKD in UK primary care practices (MacDougall et al., 1986) although this may differ with geographical location. Inhibition of RAAS in dogs with biopsy‐proven glomerular disease has been shown to be beneficial in relatively small studies (Grauer et al., 2000; Grodecki et al., 1997). RAAS inhibition is the treatment of choice for dogs with CKD and persistent proteinuria (UPC > 0.5; Brown, et al., 2013) and control of proteinuria is associated with positive outcomes in canine patients (Fulton et al., 2023).

In cats, the predominant pathology is tubulointerstitial disease (Chakrabarti et al., 2013; McLeland et al., 2015). Most cats with CKD are either non‐proteinuric or borderline proteinuric with proteinuria increasing with IRIS stage and hypertension (Syme et al., 2006). Nevertheless, proteinuria at CKD diagnosis predicts survival (Syme et al., 2006), and progression (Chakrabarti et al., 2012). Mild proteinuria in non‐azotaemic cats is associated with risk of azotaemia development (Jepson et al., 2009). RAAS inhibition reduces feline proteinuria (King et al., 2006; Sent et al., 2015) but a benefit on survival or slowing CKD progression has yet to be demonstrated. In part, this relates to the heterogeneity of CKD progression in cats with about 50% showing no progression within 12 months of diagnosis (Chakrabarti et al., 2012).

In summary both dogs and cats hyperfiltrate when nephrons are lost, in part because of upregulation of RAAS locally. Dogs demonstrate progressive pathology in the remnant kidney which is improved by lowering glomerular capillary pressure with RAAS inhibition. SGLT2 inhibitors should be synergistic with RAAS inhibitors against hyperfiltration, constricting the afferent arteriole whereas RAAS inhibitors relax the efferent arteriole to lower glomerular capillary pressure and reduce proteinuria. Reducing proteinuria in dogs is associated with improved survival and so SGLT2 inhibition could be effective by this mechanism. The evidence supporting further reduction of glomerular capillary pressure by SGLT2 inhibition is less convincing in the cat as, in the studies conducted to date (remnant kidney model), no clinically or histopathological benefit accrues from RAAS inhibition. This could be because the degree of reduction by RAAS inhibition alone is insufficient and SGLT2 inhibition in addition to RAAS inhibition is what is needed.

## 9 REDUCED RENAL TUBULAR EPITHELIAL CELL OXIDATIVE STRESS

SGLT2 inhibitors are proposed to be nephroprotective via their ability to reduce energy demands on proximal tubular epithelial cells (PTECs). This, coupled with their effects on PTEC metabolism (see below), improves the bioenergetics of this key cell whose workload and energy requirements increase considerably as an adaptive response to reduced number of functioning nephrons, leading to oxidative stress and hypoxia.

PTECs are packed full of mitochondria and actively reabsorb up to 70% of the filtered sodium load. With loss of functioning nephrons, although GFR falls globally, the remaining functioning nephrons receive a higher filtered load, as single nephron GFR increases. Furthermore, plasma proteins are increasingly pushed into the filtrate due to hyperfiltration. This places further stress on PTECs to reabsorb increasing amounts of filtered albumin, a high‐energy requiring process involving pinocytosis followed by metabolism within the endo‐lysosome (Nielsen et al., 2016).

Oxidative stress results in generation of reactive oxygen species (ROS; super oxide anion, hydroxyl radical and hydrogen peroxide) by mitochondria exceeds the capacity of the defence mechanisms to remove these (Brown, 2008). Generation of ROS is exacerbated by hypoxia and stimulated by angiotensin‐2. PTECs hypoxia is potentiated by peritubular capillary rarefaction, a process which occurs in ageing cats with CKD (Paschall et al., 2023). ROS cause damage to DNA, proteins and carbohydrates, leading to a substantial inflammatory stimulus and so are candidates for causing progressive kidney injury in CKD in humans and are postulated to be involved in dogs and cats (Jepson, 2016).

Anti‐oxidants are generally a constituent of veterinary clinical renal diets but the importance of oxidative stress in progression of naturally occurring canine and feline CKD has been relatively poorly studied. In the dog nephrectomy model, Brown (2008) demonstrated protective effects of anti‐oxidants (vitamin E, carotenoids, and lutein) fed at levels equivalent to those found in commercial renal diets on progressive loss of renal function, which were additive to those of n3‐polyunsaturated fatty acid supplementation. Evidence that oxidative stress is of importance in naturally occurring CKD in dogs and cats consists primarily cross‐sectional studies on post‐mortem tissues (Kurahara et al., 2023) or measurement of serum and/or urinary markers (Granick et al., 2021; Nishi et al., 2023) of oxidative stress comparing cats with CKD to healthy adult controls. Most of these studies were small and did not attempt to determine any associations between these markers and risk of CKD progression, hence the evidence to date is weak and mostly extrapolated from other species.

Not only do SGLT2 inhibitors reduce hyperfiltration through the TGF mechanism mentioned above, but they also reduce sodium transport via both the SGLT2 carrier and the NHE3 carrier. This means less energy expenditure in the hypertrophied remaining functioning nephrons and, as a result, reduced oxidative stress. If oxidative stress is important in naturally occurring CKD in dogs and cats (as seems likely), then SGLT2 inhibitors should have a beneficial effect through reducing tubular energy expenditure by the above mechanisms.

## 10 RENAL EPITHELIAL CELLULAR EFFECTS OF NUTRIENT EXCESS

When renal cells are exposed to nutrient excess (as in T2DM or human metabolic syndrome), SGLT2 inhibitor effects go beyond their haemodynamic and tubular transport effects to include beneficial actions on autophagy and mitophagy (see Packer, 2022 and above), phenomena explained below. Whether stimulation of autophagy is relevant to non‐diabetic veterinary patients, whose PTECs transport more glucose per nephron due to hyperfiltration, remains to be determined.

Experimental models of metabolic syndrome and diabetic nephropathy demonstrate clear upregulation of Akt/mTORC1 in PTECs and podocytes associated with progressive nephropathy (Inoki et al., 2011; Yamahara et al., 2013). In both cases, Akt/mTOR pathway inhibition ameliorated the pathology associated with nephropathy, confirming results of earlier studies with rapamycin, which reduced albuminuria and renal pathology in the leptin‐deficient obese mouse model (Mori et al., 2009).

The contention that SGLT2 functions as an energy sensor in PTECs and, under conditions of glucose excess, activated the Akt/mTOR pathway in these cells, is supported by the findings of Kogot‐Levin et al. (2020) who demonstrated Akt/mTOR upregulation in the Akita diabetic mouse model could be inhibited by dapagliflozin treatment. Elegant genetic manipulation of mTOR and SGLT2 expression proved the linkage between SGLT2 and mTOR hyperactivation in this model and the profibrotic effects of mTOR hyperactivation.

There is much interest in manipulating PGC‐1α (which is upregulated by the glitazone drugs) as a way of inhibiting renal fibrosis in human kidney diseases (diabetic and non‐diabetic; see Clark & Parikh, 2021). AMP‐kinase partners with the sirtuins in counterbalancing the Akt/mToR pathway. AMP‐kinase activity is impaired in diabetic kidney disease (humans and mouse models) and stimulation of the AMP‐kinase pathway is protective of diabetic nephropathy (Dugan et al., 2013).

Counter‐intuitively, it appears that in non‐diabetic types of CKD, remaining functioning nephrons accumulate fatty acids and glucose within their epithelial cells due to defective fatty acid metabolism. The study of metabolic disturbances in the different kidney cell types and their involvement in progressive kidney damage is in its infancy (see Miguel & Kramann, 2023). Nevertheless, the general pattern of gene ontology in human (hypertensive and diabetic) CKD patients is indicative of downregulation of fatty acid oxidation, with their key transcriptional regulation (*PPARA/PPARGC1A*) being markedly lower when compared to control patients (Kang et al., 2015). This change in metabolic regulation is associated with accumulation of lipids in PTECs, giving an apparent nutrient excess state. Similar findings were reported in different non‐diabetic mouse models of kidney fibrosis, where interventions to correct this deficiency in fatty acid oxidation abrogated the fibrosis and cellular lipid accumulation (Kang et al., 2015). Furthermore, in the rat 5/6th nephrectomy model, remnant kidneys had reduced AMP‐kinase activity and pharmacological stimulation of the AMP‐kinase pathway proved protective (Satriano et al., 2013). Thus, impairment of autophagy is a feature of various kidney diseases, not just diabetic nephropathy (Chen et al., 2021) and its activation via stimulation of AMP‐kinase and PGC1α pathways is protective against nephrotoxins, such as cisplatin (Yuan et al., 2021).

SGLT2 inhibitors mimic nutrient deprivation at the whole organism level (see above), not just at the level of PTECs. Convincing in vitro and in vivo data suggest ketone bodies inhibit the mTOR1 pathway and their production is, in part, responsible for the SGLT2‐induced nephroprotection in both high fat diet and surgical nephrectomy‐induced CKD in mice (Tomita et al., 2020). Interestingly, in these studies for empagiflozin to induce ketone body production mice had to be fasted overnight.

The fact that SGLT2 inhibitors promote erythrocytosis (Mazer et al., 2020) suggests that they exert effects via hypoxia‐inducible factors (HIFs) (see Packer, 2021). Two isoforms of HIF, HIF1α and HIF2α are upregulated by hypoxia as discussed above. HIF2α is the isoform produced by renal pericytes that influences their *EPO* production whereas HIF1α is proangiogenic and, in the diseased kidney, is proinflammatory and profibrotic (Nayak et al., 2016). By contrast, HIF2α inhibits kidney inflammation and fibrosis, particularly once CKD is established (Kong et al., 2017). Indeed, in CKD where impaired metabolism of fatty acids and glucose leads to their cellular accumulation and inhibits autophagy, HIF2α is downregulated whereas HIF1α is upregulated because of deficient AMP‐kinase and sirtuin‐1 signalling (see Packer, 2021). SGLT2 inhibitors can reverse this pattern of changes, upregulating HIF2α through upregulation of SIRT‐1 (Dioum et al., 2009; Samaan et al., 2023) and HIF1α downregulation because of reduced hypoxia and the negative effects of sirtuin‐1/AMP‐kinase on HIF1α expression (Bessho et al., 2019).

### 10.1 Do veterinary patients with CKD exhibit signs of ‘apparent nutrient excess’ in kidney tissue

The short answer to this question is we lack data at the cellular level to know but for the following reasons, the likelihood is they do. Proteinuria is associated with increased flux through the endosome/lysosome pathway leading to PTEC stress. Although the degree of proteinuria in CKD differs between dogs and cats, progressive loss of kidney function is independently associated with proteinuria in both species (Chakrabarti et al., 2012; Jacob et al., 2005), perhaps suggesting that flux of protein through the PTECs leads to apparent nutrient excess response and defective autophagy. In cats, elevated plasma phosphate is also independently associated with CKD progression (Chakrabarti et al., 2012). Similar observations have been made in dogs (Rudinsky et al., 2018). Furthermore, feeding highly available sources of inorganic phosphate gives rise to kidney damage in cats (Alexander et al., 2019). If phosphate concentration rises in the S3 segment of the PCT above a certain threshold, calciprotein particles form which are taken up into PTECs after binding to Toll‐like 4 receptors (Shiizaki et al., 2021). These particles end up in the phagolysosome and lead to deficient autophagic processing of dysfunctional organelles (Kunishige et al., 2020). Modelling experimental data from cats suggest a similar phenomenon occurs when S3 phosphate concentration exceeds 3 mmol/L (Elliott & Geddes, 2022).

Finally, reduced packed cell volume is also an independent predictor of progressive CKD in cats (Chakrabarti et al., 2012). Reduced PCV appears to be associated with increased hepcidin concentrations (Javard et al., 2017). Limited studies have been undertaken to examine the expression of the different isoforms of HIF in feline kidneys to determine whether the pattern of HIF2α suppression and HIF1α upregulation occurs in cats with CKD. Lourenço et al. (2020) demonstrated upregulation of HIF1α in cats with naturally occurring CKD compared to kidneys from healthy control cats. The HIF‐PH inhibitor, molidustat, increases red cell mass in cats with CKD suggesting suppression of HIF2α does occur (Charles et al., 2023).

Based on these observations, SGLT2 inhibitors, which mimic nutrient deprivation and upregulate pathways that promote autophagy, could reduce the profibrotic and proinflammatory signals released from PTECs. It seems reasonable to propose these signals are involved in stimulating progressive kidney pathology in cats and dogs with CKD.

What systemic metabolic changes occur in dogs and cats with naturally occurring CKD when treated with SGLT2 inhibitors remains to be determined. Experimental obese but otherwise healthy adult cats given velagliflozin (1 mg/kg PO daily for 6 weeks) showed a 2.5‐fold increase in plasma β‐hydroxybutyrate concentrations (Hoenig et al., 2018) suggesting an increase in ketone body formation occurs in healthy cats. Similarly, in a type 2 diabetes model induced in dogs by feeding a high fat diet and administering a low dose of streptozotocin to induce insulin resistance, treatment with dapagliflozin (1.25 mg/kg daily for 6 weeks), led to an increase in serum β‐hyroxybutyrate, free fatty acids and adiponectin concentrations, while lowering serum glucose concentration (Kabir et al., 2023). These changes were accompanied by upregulation of genes encoding for lipolytic pathways (subcutaneous and visceral fat) and increased AMP‐kinase activity and maximal mitochondrial respiratory function in subcutaneous fat (Kabir et al., 2023). These pre‐clinical dog and cat studies show these drugs can induce some of the metabolic changes associated with nephroprotection in human patients. The safety of inducing negative energy balance that leads to increased protein turnover in muscle to yield urea and provide glucogenic substrate for the liver warrants careful study.

## 11 CONCLUSIONS

Similarities between pathophysiological pathways leading to progression of cardiovascular and kidney diseases in human, canine and feline patients mean that SGLT2 inhibitors could provide benefits for companion animals beyond their use to control diabetes. Future clinical trials will determine whether this is the case and could well shed light on the relevance of different pathways for the beneficial effects of these drugs across species. Such trials would also need to assess the frequency of adverse events in veterinary patients with cardiovascular and kidney diseases to assess these against any benefits identified.

## AUTHOR CONTRIBUTIONS

JE and MAO contributed equally to the conceptualisation, writing – original draft and writing – review and editing of the manuscript.

## CONFLICT OF INTEREST STATEMENT

Jonathan Elliott holds consultancy contracts and research grant funding from Boehringer Ingelheim and consultancy contracts with Elanco Animal Health. Mark Oyama holds consultancy contracts and research grant funding from CEVA Animal Health.

## ANIMAL WELFARE AND ETHICS STATEMENT

As a literature (narrative) review, this manuscript does not include any primary data derived from animals or human patients that has not been presented in the peer review literature.

## Data Availability

As a literature (narrative) review, this manuscript does not include any primary data.
